# Behavioral Therapy–Based Digital Interventions for Treating Osteoarthritis: Systematic Review and Meta-Analysis

**DOI:** 10.2196/56227

**Published:** 2025-03-19

**Authors:** Beiyao Zhu, Dian Zhu, Xiao'ao Xue, Hongyi Yang, Shurong Zhang

**Affiliations:** 1 Shanghai Jiao Tong University The Ninth People's Hospital Shanghai China; 2 School of Design Shanghai Jiao Tong University Shanghai China; 3 Department of Sports Medicine Huashan Hospital Fudan University Shanghai China

**Keywords:** osteoarthritis, digital intervention, behavioral therapy, treatment, systematic review, meta-analysis, pain, impairment, quality of life, socioeconomic burden, psychotherapy-based digital intervention, patient, pain reduction

## Abstract

**Background:**

Osteoarthritis (OA) is characterized by pain, functional impairments, muscle weakness, and joint stiffness. Since OA heightens reliance on heath care resources and exacerbates socioeconomic burden, remote OA rehabilitation using digital technologies is rapidly evolving.

**Objective:**

The aim of this study was to analyze the efficacy of behavioral therapy–based digital interventions for patients with OA.

**Methods:**

This study is a systematic review of randomized controlled trials (RCTs) that assessed the effects of behavioral therapy–based digital intervention tools for OA. These RCTs were searched from inception to June 2023 in the Web of Science, Embase, Cochrane Library, Ovid, and PubMed databases.

**Results:**

Ten eligible RCTs comprising 1895 patients with OA were included. Digital tools based on either cognitive behavioral therapy (CBT) or behavior change technique (BCT) were investigated. All studies demonstrated low-to-moderate effects on pain reduction in the short term (standardized mean difference [SMD] –0.20, 95% CI –0.35 to –0.05). Six studies reported improvement in physical function (SMD –0.20, 95% CI –0.41 to 0.00), and 5 confirmed increased pain self-efficacy (SMD 0.22, 95% CI 0.02-0.42). In subgroup analysis, compared with CBT, BCT-based digital interventions demonstrated their effects on pain reduction (SMD –0.25, 95% CI –0.49 to 0.00) and physical function (SMD –0.26, 95% CI –0.54 to –0.01) in the short term. In addition, physiotherapist involvement in treatment had a positive effect on pain control (SMD –0.14, 95% CI –0.27 to –0.02). Furthermore, web-based digital tools improved physical function in the short term (SMD –0.28, 95% CI –0.54 to –0.01).

**Conclusions:**

Moderate- and low-quality evidence supported that behavioral therapy–based digital tools improved pain intensity, physical function, and self-efficacy in the short term. However, affective interactions between patients and professionals may affect the clinical outcomes.

**Trial Registration:**

PROSPERO CRD42023430716; https://tinyurl.com/yc49vzyy

## Introduction

Osteoarthritis (OA) is a prevalent joint disease affecting 29.7% of the population older than 40 years [1]. OA is characterized by pain, functional impairments, muscle weakness, joint stiffness, and reduced health-related quality of life (HRQoL) [2,3]. This causes heightened reliance on heath care resources and exacerbates socioeconomic burden [4,5]. Recent patient-centered guideline updates advocate for the implementation of rehabilitation interventions, including exercise therapy and educational programs as recommended nonpharmacological and nonsurgical treatment options [6,7]. These guidelines place a strong emphasis on the role of physical exercise, self-efficacy, and self-management strategies in the rehabilitation process for individuals with OA [8,9].

Psychological evidence–based auxiliary therapies have been integrated into the rehabilitation process to enhance rehabilitation outcomes [10]. These therapies provide a potent means of reinforcing patient education and behavior change. Behavioral theories, rooted in the social psychology of behavior change, are implemented as psychological evidence–based auxiliary therapies aimed at optimizing the positive components of interventions targeting patient health behaviors [11]. Many therapists incorporate physical therapy with behavioral therapy into their treatment protocols to alter observable maladaptive behavior patterns by eliciting new responses to given stimuli [12]. Behavioral science focuses on predicting, explaining, and altering behavior, encompassing approaches such as cognitive behavioral therapy (CBT) and behavior change techniques (BCTs) [13]. CBT, derived from the social cognitive theory, is a problem-focused approach that helps individuals identify and modify dysfunctional beliefs, thoughts, and behavior patterns contributing to their issues [14,15]. BCTs are considered active ingredients and include techniques such as feedback, self-monitoring, and behavior reinforcement. These techniques can be employed individually or in combination and in various forms [11,16]. Both CBT and BCT can enhance patients’ ability to self-manage chronic diseases, particularly among those experiencing anxiety, depression, and chronic pain [17-19]. However, study outcomes have been heterogeneous. Some have confirmed that CBT is beneficial for pain improvement [20-22] and that BCT could enhance physical activity compliance in patients with lower extremity OA [23-25], while others have argued that CBT has no effect on patients who catastrophize about pain or has only a small positive effect on pain [12,26].

The increased prevalence of OA has resulted in increasing demand for therapists [27]. To this end, remote OA rehabilitation using digital technologies (eg, telephones, websites, mobile apps) is rapidly evolving to alleviate the socioeconomic burden [28-30]. Digitalized rehabilitation is comparable to physical therapist supervision in terms of exercise quality, physical training supervision, and sport-specific self-efficacy [31,32]. Accumulating evidence has demonstrated long-term improvement in pain and physical function with digital-based rehabilitation in patients with OA [33,34]. Moreover, some studies have reported that internet-based CBT and BCT have led to significant improvements in physical activity and exercise behavior compared with traditional treatments [24,35,36].

Although several studies have investigated the impact of digital behavior change interventions based on the social cognitive theory on physical activity in patients with OA [34,37], several shortcomings remain. First, different digital tools differ in terms of the digital format and underlying behavioral theories, making it challenging to provide evidence-based recommendations for their development process. Second, existing studies have not fully explored broader outcome measures. In order to fully assess the rehabilitation outcomes of patients with OA, a wider range of outcome metrics should be considered to fully evaluate the effectiveness of digital tools based on behavioral therapies. Finally, considering that relevant studies were conducted 3-5 years ago and new digital interventions for knee OA have emerged since then, there is an urgent need for an up-to-date review and evaluation of digital intervention tools incorporating behavioral therapies. Therefore, the aim of this review was to assess the effectiveness of digital interventions based on behavioral therapies for patients with OA in terms of pain, physical function, disability, and HRQoL. In addition, subgroup analyses of digital tool formats, therapist involvement, and underlying behavioral theories were conducted.

## Methods

### Study Design

This review was performed in accordance with PRISMA (Preferred Reporting Items for Systematic Reviews and Meta-Analyses, Multimedia Appendix 1) [38] and guidelines published in the Cochrane Handbook of Systematic Evaluation [39]. This study protocol is registered with PROSPERO (International Register of Prospective Systematic Reviews: CRD42023430716).

### Eligibility Criteria

Studies fulfilling the following criteria were included in this review and meta-analysis: randomized controlled trials (RCTs); participants with knee, hip, or ankle OA; comparing CBT- or BCT-based digital interventions with other treatments; and those addressing at least one component of pain, physical function, HRQoL, pain self-efficacy, or physical activity. Only peer-reviewed studies involving participants older than 18 years were eligible for inclusion. Studies published in any language other than English and those without sufficient data were excluded. In this review, the term “digital intervention” refers to any solution or technology that delivers health information from health care providers to patients over a distance.

### Literature Search

The literature search was performed using Web of Science, Embase, Cochrane Library, Ovid, and PubMed databases, from date of inception to June 27, 2023 (Table S1 of Multimedia Appendix 2). In addition, the reference lists of relevant reviews and selected papers were manually examined for potentially relevant/eligible trials.

### Study Selection

All duplicate references were removed. Subsequently, titles and abstracts were manually screened for potentially eligible studies by 2 reviewers (DZ and HY), and relevant RCTs were identified. The full text was then retrieved by both reviewers to assess eligibility for inclusion. Disagreements between the researchers were resolved through discussion or consultation with a third reviewer (BZ). The data were cross-validated by a third researcher (BZ) using EndNote 20 (Clarivate Analytics).

### Data Extraction

Two independent reviewers (DZ and HY) collected the data. For continuous outcomes, the following data were extracted: mean (SD) and sample size at baseline and follow-up. For dichotomous outcomes, the number of cases and the total sample size were extracted. The dataset comprised study information, participant characteristics, type of behavioral therapy, type of digital tool, study duration, and outcome measures, including pain and function scores. If a study used multiple pain scales, the scale with the highest sensitivity to changes was used [40]. The function subscales of the Western Ontario and McMaster Universities Arthritis Index and the Knee Injury and Osteoarthritis Outcome Score/Hip dysfunction and Osteoarthritis Outcome Score Physical Function Shortform were used to assess functional improvement. Harmonized physical function was used, with higher scores indicating more severe physical dysfunction. The authors of studies with missing data were contacted. When the authors were unavailable, data were estimated using recommendations from the Cochrane Handbook (eg, estimation of SD from SEs) [39]. In trials in which SD was not reported, missing data were imputed from 95% CIs, SEs, *P* values, baseline changes, graphical representations, median (IQR), or SDs from baseline [41]. Trials in which imputations were not possible were excluded from the quantitative analysis.

The short-term effect was considered as follow-up period ≤6 months after randomization, and the long-term effect was considered as follow-up period >6 months after randomization. When >1 timepoint was available during the same follow-up period, the point closer to the end of the intervention was considered.

### Risk of Bias Assessment

Risk of bias was assessed using the risk-of-bias tool in the Cochrane Handbook of Systematic Evaluation [42]. Seven domains, including random sequence generation, allocation concealment, blinding of participants and personnel, incomplete outcome data, selective reporting, and other biases, were used to evaluate quality of evidence [43]. Each domain was assigned a judgement of low, high, or unclear risk of bias. Furthermore, the quality level of this meta-analysis was evaluated according to GRADE (Grading of Recommendations Assessment, Development, and Evaluation) approach [44,45]. The quality of evidence was classified as high, moderate, low, or very low.

### Data Analysis

A random-effects meta-analysis was performed using Review Manager version 5.4 (Cochrane Collaboration). Standardized mean differences (SMDs) were calculated to standardize the results to a uniform scale when studies assessed the same outcomes using different instruments. The SMDs for pain, physical function, HRQoL, pain self-efficacy, and physical activity were calculated by comparing interventions using behavioral therapy–based digital tools with other conventional methods. Therefore, a negative SMD value for pain and physical function and a positive SMD value for HRQoL, pain self-efficacy, and physical activity favor behavioral therapy–based digital tools. The magnitude of SMD was interpreted in accordance with the guidelines reported by Cohen [46], as follows: SMD <0.2 (small), 0.2-0.8 (medium), and >0.8 (large). For clinical interpretation, the mean differences were calculated.

Sensitivity and subgroup analyses were performed to assess the potential impact of the sources of heterogeneity. To investigate the potential impact of methodological quality on the estimates, we performed a sensitivity analysis by removing 1 study at a time, and trials with poor methodological quality were removed. Subgroups were defined in terms of the type of behavioral therapy (ie, CBT or BCT), type of digital tool (apps, wearable devices, and phones), and therapist involvement. The heterogeneity of the pooled studies was examined using the chi-square test and the *I*^2^ statistic, with *I*^2^>50% indicating substantial heterogeneity [47]. Publication bias was assessed by visual inspection of funnel plots and Egger test for meta-analyses ≥10 trials. All analyses were performed using Review Manager version 5.4.

## Results

### Study Selection and Characteristics

**Figure 1:**
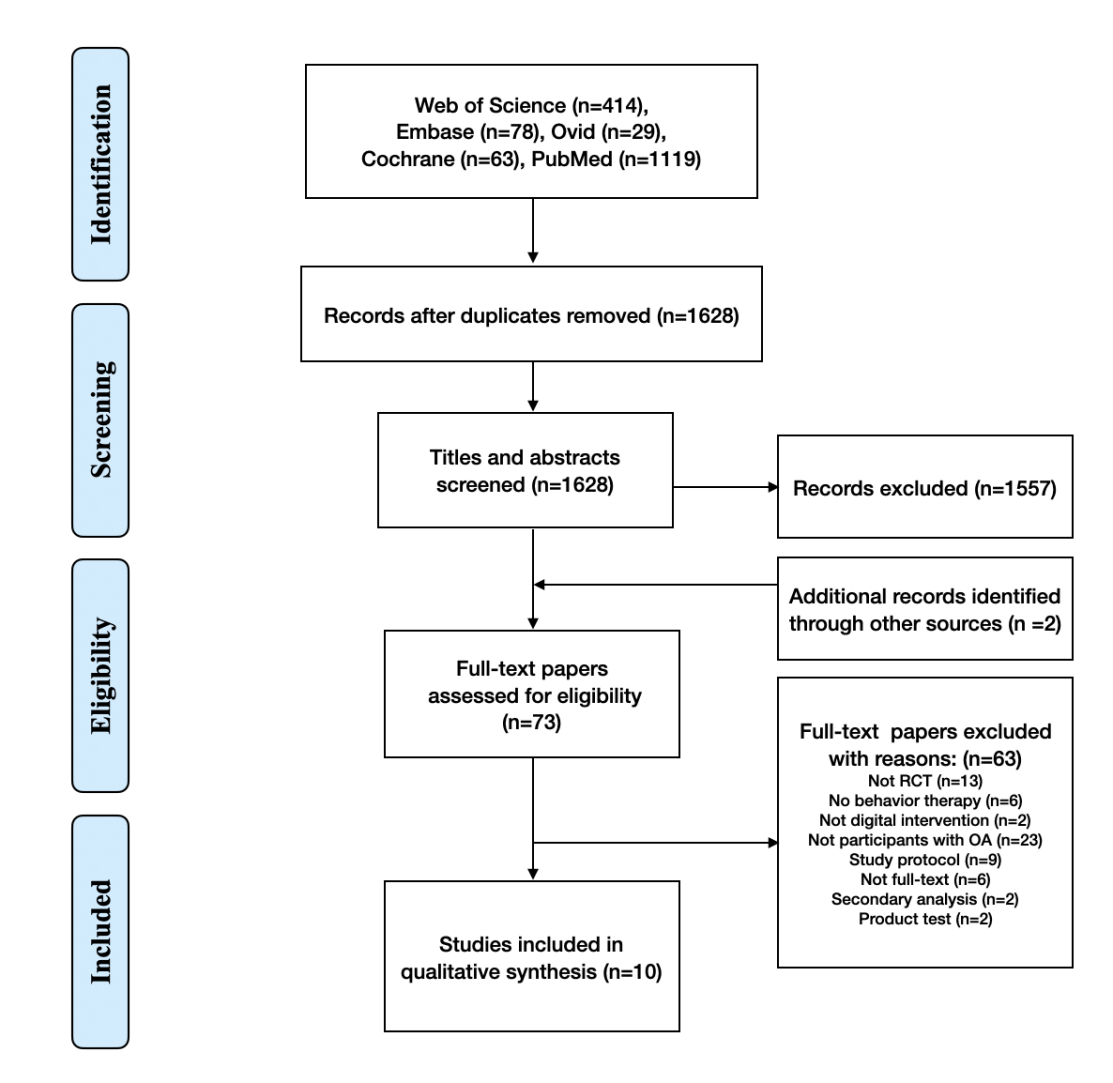
PRISMA (Preferred Reporting Items for Systematic Reviews and Meta-Analyses) flowchart of the studies selected in this review. OA: osteoarthritis; RCT: randomized controlled trial.

The initial search retrieved 1628 papers after removing duplicates, of which 1557 that did not fulfill the inclusion criteria were excluded. Two additional records were found: one through cross-referencing of bibliographies and the other by contacting the corresponding authors. Subsequently, 73 eligible full-text papers were reviewed, of which 10 RCTs were ultimately included in the quantitative analysis (Figure 1, Table 1) [24,35,36,48-53].

**Table 1:** Characteristics of the included studies.

Study author(s)	Nationality	Participants			Outcome (primary or secondary)		Follow-up (months)	Postintervention attrition (%)	
		Intervention group	Control group					Intervention group	Control group
Bennell et al [36]	Australia	SMS text message (n=56)	Non-SMS text message (n=54)	Exercise Adherence Rating Scale, days of the week to exercise (0-3), pain (Knee Injury and Osteoarthritis Outcome Score), overall mean knee pain (Numeric Rating Scale), symptoms (Knee Injury and Osteoarthritis Outcome Score), function (Knee Injury and Osteoarthritis Outcome Score), knee-related quality of life (Knee Injury and Osteoarthritis Outcome Score), health-related quality of life, self-efficacy: pain (Arthritis Self-Efficacy Scale), self-efficacy: function (Arthritis Self-Efficacy Scale), self-efficacy: other (Arthritis Self-Efficacy Scale), Behavioral Factors in Osteoarthritis Management Scale, Physical Activity Scale for the Elderly, Pain Catastrophizing Scale		6		14.29	5.55
Rini et al [48]	United States	Digital application (n=58)	Nonintervention (n=55)	Pain, self-efficacy in pain management, pain-related anxiety, pain-related functional interference, positive and negative emotions		3		1.72	5.45
Hinman et al [49]	Australia	Campaign advice and support (n=87)	Available services (n=88)	Overall mean knee pain (Numeric Rating Scale), Pain with Activities of Daily Living (Western Ontario and McMaster Universities Arthritis Index), mean pain while walking (Numeric Rating Scale), Pain Self-Efficacy (Arthritis Self-Efficacy Scale), Functional Self-Efficacy (Arthritis Self-Efficacy Scale), Fear of Movement, Physical Activity Scale for the Elderly, barriers to physical activity, benefits of physical activity, health-related quality of life		6		5.74	13.63
McCurry et al [50]	United States	Cognitive behavioral therapy-I (n=163)	Education (n=164)	Insomnia Severity Index, Flinders Fatigue Scale, Arthritis Pain Intensity and Interference with Activity, Brief Pain Inventory-short form, Patient Health Questionnaire-8 items, Measure of Depression		12		14.72	6.09
Pelle et al [26]	Netherlands	Dr Bart (n=214)	Conventional physiotherapy group (n=213)	Incidence rate ratio, pain, symptoms and dysfunction (Knee Injury and Osteoarthritis Outcome Score or Hip dysfunction and Osteoarthritis Outcome Score), quality of life (EQ-5D-3L), total hours of physical activity, Patient Activation Measure-13 items, knowledge skills confidence, Illness Perception Questionnaire cognitive and emotional perceptions		6		39.25	22.07
Mecklenburg et al [51]	United States	Hinge Health (n=101)	Education (n=61)	Pain, Physical Function Scale (Knee Injury and Osteoarthritis Outcome Score), Visual Analog Scale Pain, Visual Analog Scale Hardness Score, surgical intent		3		33.33	40.98
Nelligan et al [24]	Australia	Automatic Behavior Change SMS Support (n=103)	Web-based (n=103)	Physical Function (Western Ontario and McMaster Universities Osteoarthritis Index), Overall Mean Knee Pain (Numeric Rating Scale), Pain, Knee Quality of Life, Sport and Recreation (Knee Injury and Osteoarthritis Outcome Score), Assessment of quality of life-6D Physical Activity Scale, Arthritis Self-Efficacy Scale, Exercise Self-Efficacy Scale		6		11.65	10.68
Bennell et al [35]	Australia	Telephone counseling (n=84)	Nontelephone counseling (n=84)	Pain, physical function (Western Ontario and McMaster Universities Osteoarthritis Index), Numeric Rating Scale walking pain (range 0-10), Assessment of quality of life II, Physical Activity Scale for the Elderly, Athletic Activity Scale total activity time, step time (hours/day)		18		14.29	16.67
Kloek et al [52]	Netherlands	Web-based (n=109)	Conventional physiotherapy group (n=99)	Physical functioning, functions of daily living, functional limitations (Hip dysfunction and Osteoarthritis Outcome Score or Knee Injury and Osteoarthritis Outcome Score), self-perceived effects, arthritis self-efficacy scale, pain, and fatigue		12		18.30	12.10
Li et al [53]	Canada	Fitbit (immediate group) (n=26)	Education (delay group) (n=25)	Mean daily moderate to vigorous physical activity, Knee Injury and Osteoarthritis Outcome Score, Partners in Health Scale, Theory of Planned Behavior Questionnaire, Patient Health Questionnaire-9 items, Self-Reporting Habits Index		9.75		7.70	4

### Study Characteristics

In this qualitative analysis, 9 RCTs and 1 cluster RCT, comprising 1895 patients, were included (Tables S2-S4 [24,26,35,36,48-53] of Multimedia Appendix 2). The trials were conducted in Europe (2/10, 20%) [26,52], Oceania (4/10, 40%) [24,35,36,49], and North America (4/10, 40%) [48,50,51,53]. The sample sizes of the included trials ranged from 51 to 427; the mean age of the patients was 62.5 (SD 8.1) years, and females were predominant in the pooled population (1231/1895, 64.9%), which is consistent with the global prevalence of OA. Six studies recruited participants with knee OA only [24,35,36,49,51,53], whereas 4 recruited participants with both knee and hip OA [26,48,50,52]. CBT-based (4 studies) [35,48,50,51] and BCT-based (6 studies) [24,26,36,49,52,53] therapies were the most used behavioral therapies blended into the digital intervention for patients with OA. Three types of digital tools were used: apps or websites in 9 studies [24,26,35,36,48,49,51-53], wearable devices in 3 studies [51-53], and SMS text messages in 1 study [50]. Four studies included face-to-face communication with physical therapists or fitness instructors [36,51-53]. All trials reported short-term effects (up to 6 months after randomization) [24,26,35,36,48-53] and 6 reported long-term effects (>6 months after randomization) [24,26,35,50,52,53]. Pain intensity, physical function, HRQoL, pain self-efficacy, and physical activity were evaluated in 10, 6, 6, 5, and 5 trials, respectively.

### Risk of Bias in the Included Trials

**Figure 2:**
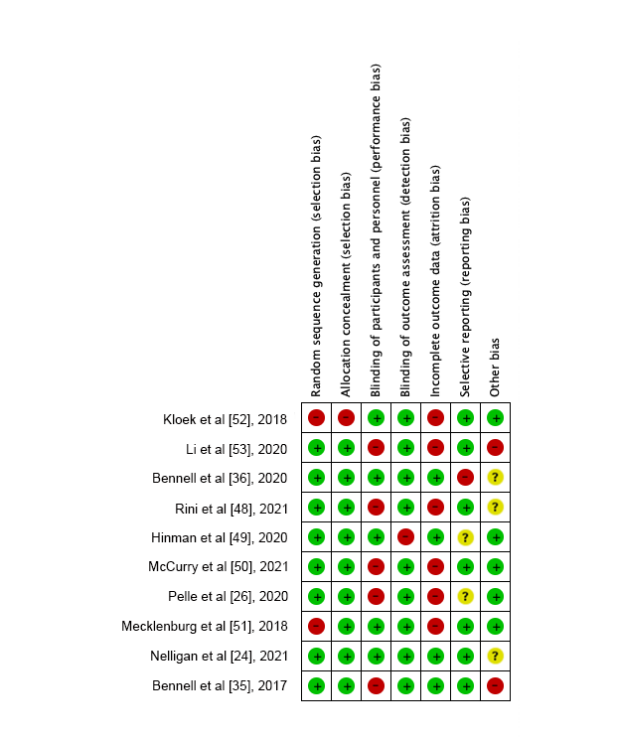
Risk of bias. Red indicates high risk, green indicates low risk, and yellow indicates unclear risk.

In general, the most frequent risks of bias for RCTs were incomplete outcomes (6/10, 60%) and blinding of participants/personnel (6/10, 60%). Other biases and selective reporting accounted for the second most frequent risks of bias (2/10, 20%). The overall confidence in the cumulative evidence varied from very low to moderate, with low confidence being the most commonly identified (Figure 2 [24,26,35,36,48-53], Table 2).

**Table 2:** Summary of the findings for efficacy outcomes.^a^

Outcome, time			Studies (patients), n	SMD^b^ (95% CI)	*I*^2^ (%)	Quality of evidence assessment					
						Risk of bias	Inconsistency	Indirectness	Imprecision	Publication bias	Rating^c^
Pain (short-term)			10 (1895)	–0.20 (–0.35 to 0.05)	65	–^d^	–	+^e^	+	+	Low
Pain (long-term)			6 (1271)	–0.01 (–0.12 to 0.10)	0	–	+	+	+	+	Moderate
Physical function (short-term)			6 (974)	–0.20 (–0.41 to 0.00)	62	–	–	+	+	+	Low
Physical function (long-term)			3 (519)	–0.13 (–0.32 to 0.06)	0	–	+	+	+	+	Moderate
Health-related quality of life (short-term)			6 (1137)	0.28 (–0.05 to 0.61)	86	–	–^–2^^f^	+	+	+	Very low
Pain self-efficacy (long-term)			5 (812)	0.22 (0.02 to 0.42)	10	–	+	+	–	+	Low
Physical activity (short-term)			5 (1086)	0.05 (–0.07 to 0.17)	0	–	+	+	+	+	Moderate
**Subgroup outcome**											
	**Behavior therapy pain outcomes (short-term)**										
		BCT^g^	6 (1137)	–0.25 (–0.49 to 0.00)	74	–	–	+	+	+	Low
		CBT^h^	4 (758)	–0.10 (–0.24 to 0.05)	0	–	+	+	+	–	Low
	**Behavior therapy pain outcomes (long-term)**										
		BCT	4 (781)	–0.01 (–0.15 to 013)	0	–	+	+	+	+	Moderate
		CBT	2 (490)	0.00 (–0.18 to 0.17)	0	–	+	+	+	+	Moderate
	**Physical function outcomes (short-term)**										
		BCT	5 (659)	–0.28 (–0.54 to –0.01)	67	–	–	+	+	+	Low
		CBT	2 (363)	–0.05 (–0.26 to 0.16)	0	–	+	+	+	–	Low
	**Physical function outcomes (long-term)**										
		BCT	2 (311)	–0.21 (–0.44 to 0.02)	6	–	+	+	+	–	Low
		CBT	1 (208)	0.00 (–0.28.0.27)	N/A^i^	–	+	+	+	–	Low
	**Pain outcomes with therapist involvement (short-term)**										
		With	6 (1039)	–0.14 (–0.27 to –0.02)	0	–	+	+	+	+	Moderate
		Without	4 (856)	–0.23 (–0.60 to 0.14)	85	–	–^–2^	+	+	+	Very low
	**Pain outcomes with therapist involvement (long-term)**										
		With	5 (844)	–0.04 (–0.17 to 0.10)	0	–	+	+	+	+	Moderate
		Without	1 (427)	0.05 (–0.14 to 0.24)	N/A	–	+	+	+	–	Low
	**Physical function outcomes with therapists (short-term)**										
		With	4 (706)	–0.17 (–0.40 to 0.07)	60	–	–	+	+	+	Low
		Without	2 (316)	–0.27 (–0.75 to 0.20)	76	–	–	+	+	+	Low
	**Pain outcomes with digital tools (short-term)**										
		Telephone	1 (282)	–0.09 (–0.33 to 0.14)	N/A	–	+	+	+	–	Low
		Web-based	6 (1199)	–0.23 (–0.47 to 0.01)	75	–	–^–2^	+	+	+	Very low
		Wearable	3 (414)	–0.12 (–0.31 to 0.08)	0	–	+	+	+	–	Low
	**Pain outcomes with digital tools (long-term)**										
		Telephone	1 (282)	–0.03 (–0.27 to 0.20)	N/A	–	+	+	+	–	Low
		Web-based	3 (738)	–0.02 (–0.16 to 0.12)	0	–	+	+	+	+	Moderate
		Wearable	2 (251)	0.05 (–0.20 to 0.30)	0	–	+	+	+	–	Low
	**Physical function outcomes with digital tools (short-term)**										
		Web-based	4 (659)	–0.28 (–0.54 to –0.01)	67	–	–	+	+	+	Low
		Wearable	2 (363)	–0.05 (–0.26 to 0.16)	0	–	+	+	+	–	Low
	**Physical function outcomes with digital tools (long-term)**										
		Web-based	2 (311)	–0.21 (–0.44 to 0.02)	6	–	+	+	+	–	Low
		Wearable	1 (208)	0.00 (–0.28 to 0.27)	N/A	–	+	+	+	–	Low

^a^A funnel plot and Egger test was used to judge publication bias (Figure S1 of Multimedia Appendix 2).

^b^SMD: standardized mean difference.

^c^GRADE (Grading of Recommendations Assessment, Development, and Evaluation) Working Group grades of evidence. High: further research is very unlikely to change our confidence in the estimate of effect; moderate: further research is likely to have an important impact on our confidence in the estimate of effect and may change the estimate; low: further research is very likely to have an important impact on our confidence in the estimate of effect and is likely to change the estimate; very low: we are very uncertain about the estimate.

^d^– indicates low-quality evidence.

^e^+ indicates high-quality evidence.

^f^–^–2^ indicates a moderate to high degree of inconsistency.

^g^BCT: behavior change technique.

^h^CBT: cognitive behavioral therapy.

^i^N/A: not applicable. Since only 1 study was included, there was no heterogeneity in the analysis.

### Main Outcomes

**Figure 3:**
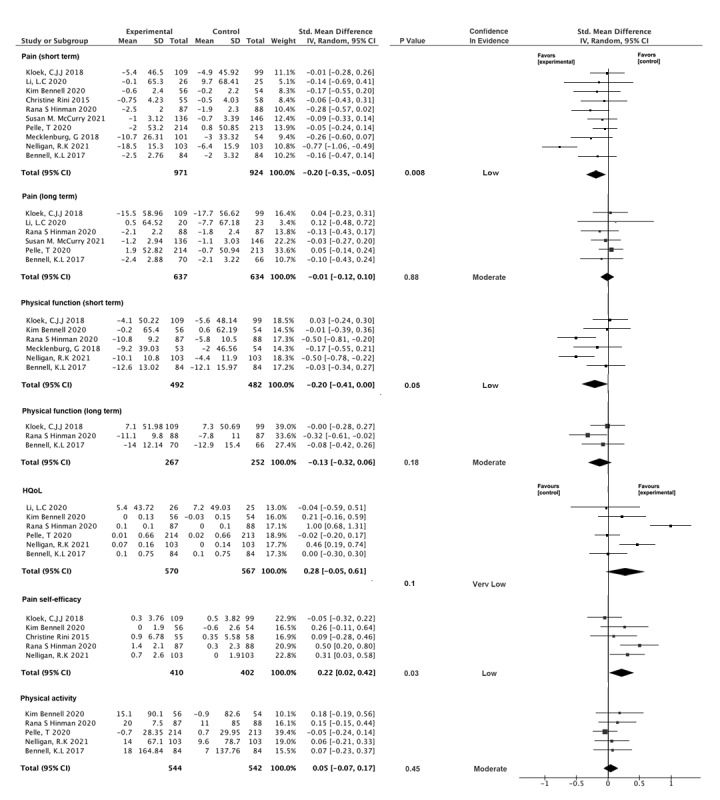
Main results of this study. HQoL: health-related quality of life.

For pain reduction, 10 trials with 1895 patients reported a moderate effect of behavioral therapy–based digital tools in reducing pain in the short term (4-24 weeks) (SMD –0.20, 95% CI –0.35 to –0.05; *P*=.008; *I*^2^=58%), with low-quality evidence [24,26,35,36,48-53]. However, in the long-term follow-up (≥24 weeks), this effect was ambiguous when 6 trials with 1271 patients were included (SMD –0.01, 95% CI –0.12 to 0.10; *P*=.88; *I*^2^=0%), with moderate-quality evidence [26,35,49,50,52,53]. For physical function, low certainty evidence supported significant improvement in the short term (SMD –0.20, 95% CI –0.41 to 0.00; *P*=.05; *I*^2^=62%) from 6 trials with 974 patients [26,35,36,49,50,52]. However, its effect showed no significance in 3 trials with 519 patients in the long term (SMD –0.13, 95% CI –0.32 to 0.06; *P*=.18; *I*^2^=17%), with moderate-quality evidence [35,49,52]. Sensitivity analysis revealed that the results of physical function were unstable; therefore, these results should be interpreted with caution. For pain self-efficacy, low-quality evidence from 5 trials with 812 patients reported a positive effect (SMD 0.22, 95% CI 0.02-0.42; *P*=.03; *I*^2^=10%) [24,36,48,49,52]. However, for HRQoL, very low-quality evidence from 6 trials with 1137 patients demonstrated an uncertain effect (SMD 0.28, 95% CI –0.05 to 0.61; *P*=.10; *I*^2^=86%) [24,26,35,36,49,53]. Further, moderate quality evidence demonstrated an uncertain effect (SMD 0.05, 95% CI –0.07 to 0.17; *P*=.45; *I*^2^=0%) for physical activity [24,26,35,36,49]. All results are presented in Figure 3 [24,26,35,36,48-53].

### Subgroup Analyses

Subgroup analyses of behavioral therapy, digital tools, and therapist involvement were performed, and significant improvements were found in the short-term intervention.

#### Behavioral Therapy

**Figure 4:**
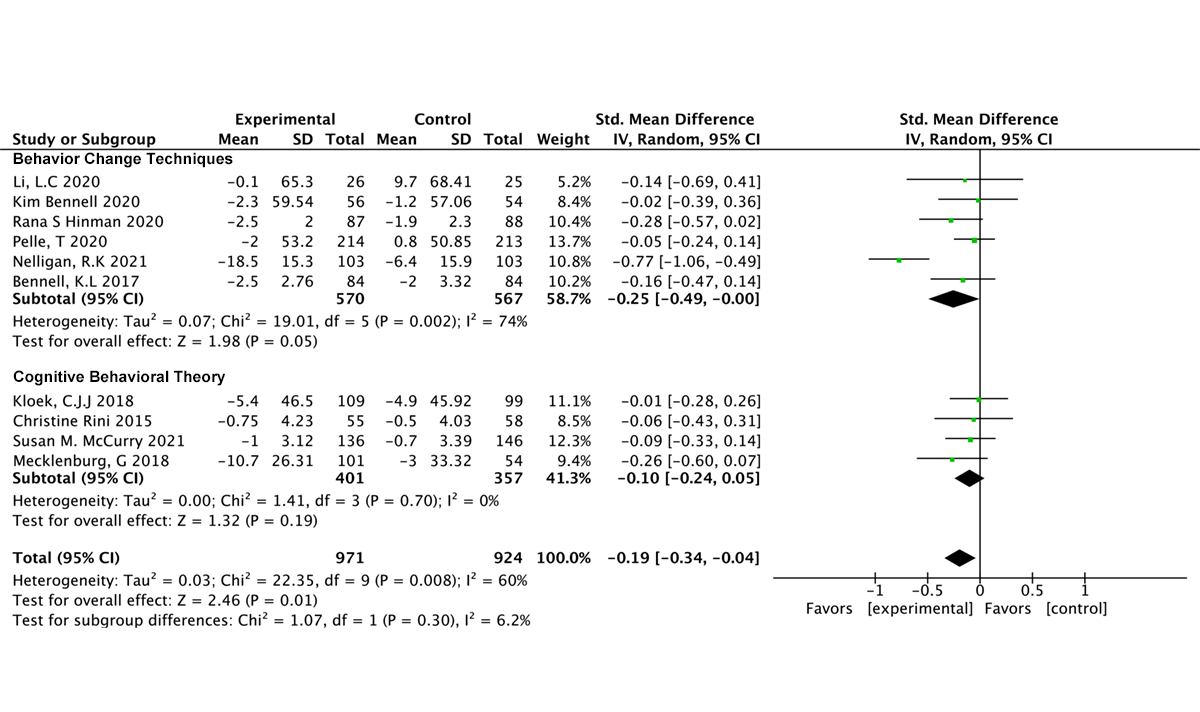
Pain outcomes in different behavioral therapy models.

BCTs (6 studies, 1177 participants) demonstrated a significant impact on pain reduction (SMD –0.25, 95% CI –0.49 to 0.00; *P*=.05; *I*^2^=74%) [24,26,35,36,49,53]. However, our findings indicate that CBT (n=4 studies, n=758 participants) did not have a significant effect on pain (SMD –0.10, 95% CI –0.24 to 0.05; *P*=.19; *I*^2^=0%) [48,50-52] (Figure 4 [24,26,35,36,48-53]).

**Figure 5:**
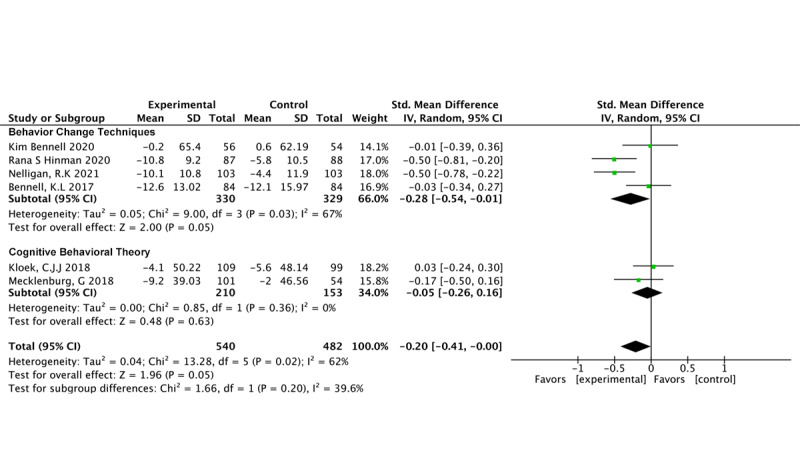
Physical function outcomes in different behavioral therapy models.

BCTs (4 studies, 659 participants) were found to significantly reduce physical functional impairment (SMD –0.28, 95% CI –0.54 to –0.01; *P*=.05; *I*^2^=67%) [24,35,36,49]. In contrast, our findings show that CBT (n=2 studies, n=363 participants) did not significantly affect pain (SMD –0.20, 95% CI –0.26 to 0.16; *P*=.63; *I*^2^=0%) [51,52] (Figure 5 [24,35,36,49,51,52]).

#### Therapist Involvement

**Figure 6:**
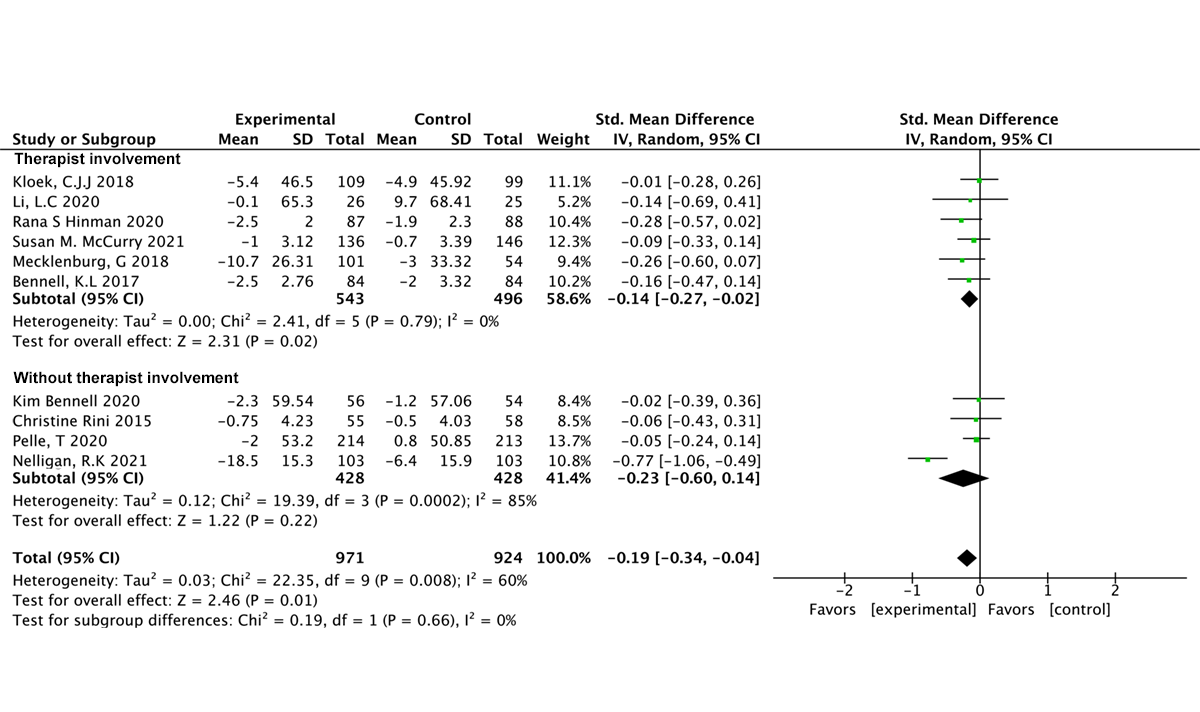
Pain outcomes related to therapist involvement.

Six studies involving 1039 participants revealed significant effects favoring the intervention in pain reduction when therapist involvement was present (SMD –0.14, 95% CI –0.27 to –0.02; *P*=.02; *I*^2^=0%) [35,49-53]. Conversely, 4 studies involving 856 participants revealed no significant effects in pain reduction for interventions without therapist involvement (SMD –0.23, 95% CI –0.60 to 0.14; *P*=.22; *I*^2^=85%) [24,26,36,48] (Figure 6 [24,26,35,36,48-53]).

**Figure 7:**
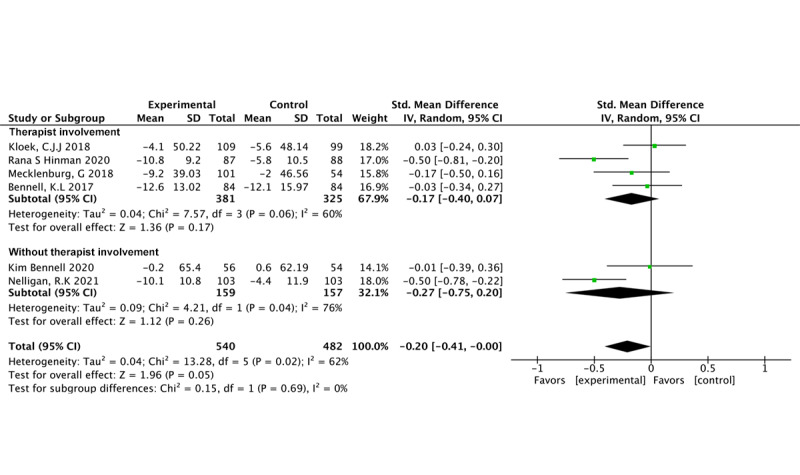
Physical function outcomes related to therapist involvement.

Four studies involving 706 participants (SMD –0.17, 95% CI –0.40 to 0.07; *P*=.17; *I*^2^=60%) [35,49,51,52] and 2 studies involving 316 participants [24,36] (SMD –0.22, 95% CI –0.37 to –0.06; *P*=.26; *I*^2^=76%) revealed that therapist involvement did not result in significant improvements in physical functional impairment (Figure 7 [24,35,36,49,51,52]).

#### Digital Tools

**Figure 8:**
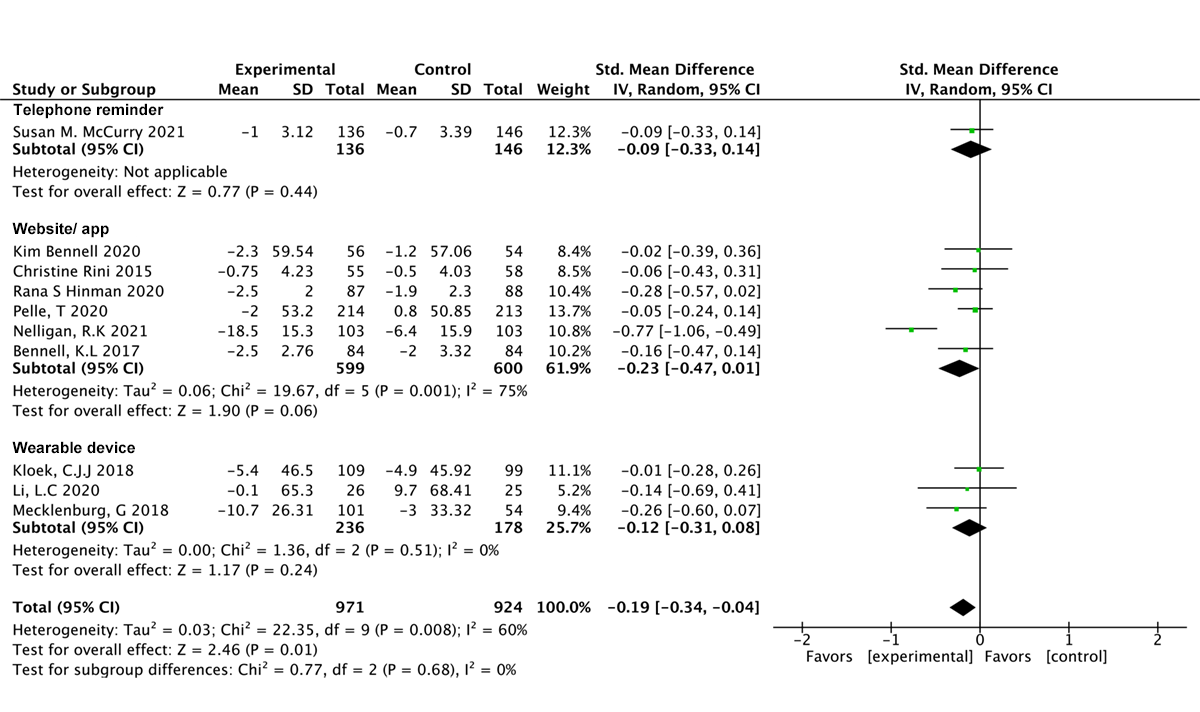
Pain outcomes with different digital tools.

Neither the use of a mobile app/website (SMD –0.23, 95% CI –0.47 to 0.01; *P*=.06; *I*^2^=75%) [50], telephone communication (SMD –0.09, 95% CI –0.33 to 0.14; *P*=.44) [24,26,35,36,48,49] nor the use of a wearable device as a monitoring device (SMD –0.12, 95% CI –0.31 to 0.08; *P*=.24; *I*^2^=0%) [51-53] were associated with the effect in favor of the intervention (Figure 8 [24,26,35,36,48-53]).

**Figure 9:**
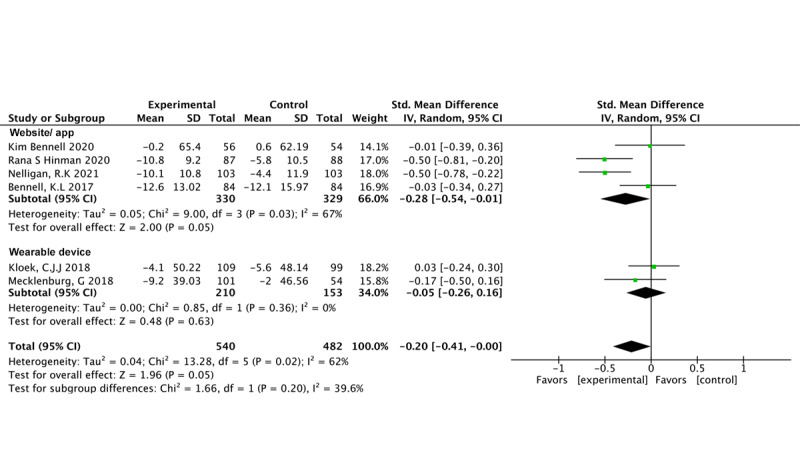
Physical function outcomes with different digital tools.

Four studies involved 659 participants who used a mobile app/website and observed a significant effect in favor of the intervention in terms of physical dysfunction (SMD –0.28, 95% CI –0.54 to –0.01; *P*=.05; *I*^2^=67%) [24,35,36,49]. However, there was no difference for only 2 items involving wearables (SMD –0.05, 95% CI –0.26 to 0.16; *P*=.63; *I*^2^=0%) [51,52] (Figure 9 [24,35,36,49,51,52]).

### Risks of Bias Sensitivity Analysis

After excluding studies at high risk of bias, the results regarding randomization, allocation concealment, incomplete outcome reporting, selective reporting, and other biases demonstrated stability. However, instability was observed in the results when studies with unclear patient blinding were excluded. For outcomes related to physical function, studies excluding those at high risk of bias showed stability in allocation concealment and incomplete outcome reporting. Nevertheless, exclusion of trials with biases related to randomization, blinding, selective reporting, and other factors resulted in unstable findings. Therefore, these results warrant cautious interpretation (Table 3). Additionally, due to the inclusion of predominantly fewer than 10 studies, the potential impact of small-study effects was explored using only funnel plots. The inspection of funnel plots (Figure S1 of Multimedia Appendix 2) suggested small study effects/publication bias for the overall results in terms of pain in the short and medium terms.

**Table 3:** Sensitivity of the studies.

Variable				Number of studies (patients)		Standardized mean difference (95% CI)		*I*^2^ (%)		Overall effect *P* value		Subgroup differences *P* value	
**Pain**													
	**Randomization**												.52
		High risk and unclear risk	2 (363)		–0.12 (–0.37 to 0.13)		26		.34				
		Low risk	8 (1532)		–0.22 (–0.40 to –0.04)		65		.02				
	**Allocation concealment**												.18
		High risk and unclear risk	1 (208)		–0.01 (–0.28 to 0.26)		0		.94				
		Low risk	9 (1687)		–0.22 (–0.38 to –0.06)		60		.006				
	**Blinding**												.30
		High risk and unclear risk	6 (1216)		–0.11 (–0.23 to 0.00)		0		.05				
		Low risk	4 (679)		–0.31 (–0.66 to 0.05)		81		.09				
	**Incomplete outcome**												.14
		High risk and unclear risk	5 (1123)		–0.09 (–0.20 to 0.03)		0		.15				
		Low risk	5 (772)		–0.30 (–0.57 to –0.04)		70		.03				
	**Selective reporting**												.37
		High risk and unclear risk	4 (825)		–0.12 (–0.25 to 0.02)		0		.09				
		Low risk	6 (1070)		–0.25 (–0.49 to 0.00)		73		.05				
	**Other bias**												.22
		High risk and unclear risk	4 (535)		–0.34 (–0.68 to 0.01)		73		.06				
		Low risk	6 (1360)		–0.11 (–0.21 to –0.01)		0		.05				
**Physical function**													
	**Randomization**												.20
		High risk and unclear risk	4 (659)		–0.28 (–0.54 to –0.01)		67		.05				
		Low risk	2 (363)		–0.05 (–0.26 to 0.16)		0		.63				
	**Allocation concealment**												.10
		High risk and unclear risk	1 (208)		0.03 (–0.24 to 0.30)		N/A^a^		.83				
		Low risk	5 (814)		–0.26 (–0.47 to –0.04)		58		.02				
	**Blinding**												.71
		High risk and unclear risk	3 (343)		–0.27 (–0.73 to 0.19)		79		.25				
		Low risk	3 (679)		–0.17 (–0.43 to 0.09)		63		.19				
	**Incomplete outcome**												.20
		High risk and unclear risk	2 (363)		–0.05 (–0.26 to 0.16)		0		.63				
		Low risk	4 (659)		–0.28 (–0.54 to –0.01)		67		.05				
	**Selective reporting**												.71
		High risk and unclear risk	2 (285)		–0.27 (–0.75 to 0.21)		75		.27				
		Low risk	4 (737)		–0.17 (–0.41 to 0.07)		64		.17				
	**Other bias**												.94
		High risk and unclear risk	3 (484)		–0.20 (–0.52 to 0.13)		69		.25				
		Low risk	3 (538)		–0.21 (–0.53 to 0.11)		70		.19				

^a^N/A: not applicable. Since only 1 study was included, there was no heterogeneity in the analysis.

## Discussion

### Main Findings

This systematic review and meta-analysis report provides moderate- to low-quality evidence regarding the therapeutic effects of 3 digital tools based on 2 behavioral therapies for patients with OA. Digital tools focusing on behavior change have shown short-term efficacy, including reductions in pain intensity and physical function impairment along with improvements in pain self-efficacy. However, our study did not find significant differences in sports and recreational activities or overall physical activity. Given the risk of bias in the included studies, the overall quality of evidence is moderate to low. Furthermore, the evidence quality regarding HRQoL outcomes is deemed very low; so, the findings should be interpreted with caution.

Our study compares the short-term and long-term effects of behavior theory–based digital interventions on pain. However, contrary to Safari et al’s [34] previous findings, we found no significant long-term effects of behavior theory–based digital interventions. This discrepancy may stem from the incomplete success of digital interventions in promoting adherence to prescribed exercise regimens among patients with musculoskeletal disease [30,54]. A recent review indicated that digital interventions have not been shown to enhance compliance with therapeutic exercise among patients with chronic musculoskeletal disease [30]. Therefore, further high-quality research is needed to conclusively determine the efficacy of these interventions for long-term therapeutic effects.

Among the 10 studies included, 9 observed positive changes in patient pain [24,26,35,36,48-51,53]. Current research integrates behavior therapy principles into digital interventions aimed at enhancing patients’ ability to modify their decision-making frameworks and guide behavior change in patients with OA [55]. The educational components of digital tools include pathophysiological and etiological information on OA, guideline-based therapies, exercise for OA management, and pain management strategies, and these are closely aligned with the social cognitive theories of health behavior [19]. These address motivational and volitional determinants of exercise behavior [31,56]. Our study suggests that such semisupervised, freely accessible interventions may be an effective option for reducing pain [24], as pain intensity correlates positively with the use of exercise, courses, and health self-management messaging functionalities [57]. Additionally, improvements in physical function impairment were observed in 6 studies. Research has demonstrated the impact of digital health interventions on physical function through exercise and aerobic training programs [58,59]. This is because the digital tools included in the studies mostly incorporate multicomponent exercises recommended by clinical guidelines, which can enhance balance and mobility [60,61].

Low-quality evidence revealed that a behavioral therapy–based digital tool had a positive effect on pain self-efficacy, which is consistent with previous research that found greater self-confidence activation in digital self-monitoring programs than in traditional management programs [33,62,63]. Self-efficacy refers to an individual’s perception of being capable of making positive changes in their lives can help them to be more likely to initiate and maintain positive behavior changes [64,65]. Previous studies have reported significant indirect effects of digital tools on health behaviors, and we suggest that self-efficacy could be used as an indirect mediator in clinical practice [65,66].

This review indicates that behavior therapy–based digital interventions have no significant impact on HRQoL, which is consistent with that reported in a previous systematic review [67]. However, the mechanisms underlying patients’ perceptions of quality of life remain unclear, necessitating validated behavior change measures such as patient activation measures to effectively assess such systems. Additionally, we found that behavior therapy–based digital interventions do not significantly influence physical activity levels [55,68]. This may be attributed to the lack of face-to-face supervision. Nonetheless, despite these findings, such interventions could potentially offer substantial public health benefits, despite the modest effect size.

### Subgroup Findings

The effects of behavioral therapy were independently assessed in a subanalysis. However, our results were not consistent with those of previous studies in that only BCT-based digital tools had an impact on outcomes in patients with OA [16,20,33]. In previous studies, it was difficult to trace patient participation on a digital platform, and multiple variations could be introduced into the outcomes [69,70]. The underlying mechanism by which behavior change–based digital tools can reduce pain and physical function in patients with OA is that such interventions can elicit patient intentions and modify their decision-making structures [55]. The educational components of the digital tools include information regarding the pathology and etiology of OA, treatment according to guidelines, exercise for OA, and pain and symptom relief, which are closely related to the social cognitive theories of health behavior [19]. These factors examine the influences of motivation and willpower on exercise behavior [31,56]. Our research indicated that this semisupervised, freely accessible intervention may be effective for pain reduction [24].

Another interesting finding in our study was that physical therapist involvement was shown to be effective in pain reduction and physical function, although with low-quality evidence. This is consistent with previous evidence suggesting that communication and interaction with professionals can increase patient motivation [67,71]. Digital tools provide the opportunity to assume the role of an aid to assist patients in engaging in less complex exercises as part of independent home training [72,73]. However, it sometimes cannot replace physical therapists who achieve similar effects [31,74,75]. Typical digital intervention tools rely on self-management and lack timely reminders and professional communication [67,71]. Studies have shown that digital tools without therapist involvement do not offer significant advantages over conventional care interventions [26]. Therefore, future digital intervention tools should aim to blend traditional OA care with digital OA care solutions. Heath care should provide solutions for personalized, comprehensive, simple, reliable, and continuous mixed heath care [76,77].

Another finding is that digital tools based on website or application development show significantly better short-term improvements in physical function compared to wearable devices. This may be due to the complex wearing process of wearable devices, especially among older adults who may have resistance to wearing such devices and find them potentially hindering daily activities. Hence, future application development should fully consider patient characteristics and needs rather than solely relying on hospital staff knowledge and experience [78,79].

### Clinical and Research Implications

This study confirms that digital tools combined with behavioral therapy, especially BCTs, have moderate and clinically meaningful treatment effects in patients with hip and knee OA in the short and long term. These findings echo the current guidelines that recommend rehabilitation, emphasizing exercise, self-efficacy, and self-management for patients with OA. As digital technologies rapidly evolve, the affective interaction between patients and professionals remains a crucial factor and should be considered in future studies.

### Strengths and Limitations

This study has several limitations. First, we were unable to fully explore the reasons for heterogeneity because many covariates for behavioral therapy–based digital tools in OA were not usually reported in the trials. Second, subgroup analyses to explore the potential impact of a high risk of bias and characteristics of the population were limited by the small number of included trials or because the data were poorly reported. Third, despite the use of extensive search techniques, the evaluation may have been hampered by language bias because we only included studies published in English in the 5 databases. Therefore, we cannot guarantee that all potentially eligible studies were included. Fourth, this meta-analysis included only 10 studies, with 9 RCTs and 1 RCT cluster. Owing to this rather low number of studies, the Q-static was reduced in power, and *I*^2^ could have been biased. Finally, this study was prone to publication bias and other risks of bias and was limited only to the information reported in the studies.

### Conclusions

We found moderate- and low-quality evidence supporting behavioral therapy–based digital tools improving pain intensity, physical function, and self-efficacy in the short term. Low- and very-low-quality evidence demonstrated uncertain effects of physical activity on HRQoL or its long-term effects. However, affective interactions between patients and professionals may affect clinical outcomes. Our findings should be evaluated by clinicians, stakeholders, and researchers, considering that most digital tools currently have low or very low certainty of evidence. Our findings highlight the need to conduct large-scale trials with high methodological quality.

## Data Availability

Data are available from the corresponding author on reasonable request.

## Appendix

Multimedia Appendix 1 PRISMA checklist.

Multimedia Appendix 2 Supplementary data, including search strategy, demographic data of the study participants, mechanisms for implementing the research intervention, functions of digital tools, and funnel plot for pain.

## Abbreviations

BCT: behavior change technique
CBT: cognitive behavioral therapy
GRADE: Grading of Recommendations Assessment, Development, and Evaluation
HRQoL: health-related quality of life
OA: osteoarthritis
PRISMA: Preferred Reporting Items for Systematic Reviews and Meta-Analysis
PROSPERO: International Register of Prospective Systematic Reviews
RCT: randomized controlled trial
SMD: standardized mean difference
