# Supplementary material for: Behavioral Therapy–Based Digital Interventions for Treating Osteoarthritis: Systematic Review and Meta-Analysis
Source: J Med Internet Res. 2025 Mar 19;27:e56227. doi: 10.2196/56227 (PMC11966084; doi:10.2196/56227)
Supplement: Multimedia Appendix 1 [file jmir_v27i1e56227_app1.docx]

| **Section and Topic** | **Item #** | **Checklist item** | **Location where item is reported** |
| --- | --- | --- | --- |
| **TITLE** | | |  |
| Title | 1 | Psychotherapy-Based Digital Intervention for Treating Osteoarthritis: A Systematic Review and Meta-analysis |  |
| **ABSTRACT** | | |  |
| Abstract | 2 | **Objective**  To analyse the efficacy of psychotherapy-based digital intervention(s) in patients with osteoarthritis (OA).  **Design**  Systematic review of randomized controlled trials (RCTs).  **Data sources**  The Web of Science, Embase, Cochrane Library, Ovid, and PubMed databases were searched from inception to June 2023.  **Eligibility criteria**  RCTs that assessed the effects of psychotherapy-based digital intervention tools on OA.  **Results**  Eleven eligible RCTs comprising 2018 patients with OA were included. Three digital tools based on either cognitive behavioural therapy (CBT) or the behavioural change technique (BCT) were investigated. All studies demonstrated low to moderate effects on pain reduction in the short term (standardised mean difference [SMD] -0.18 [95% CI -0.33 to -0.04) and long term (SMD -0.18 [95% CI -0.33 to -0.04]). Eight studies reported improvement in physical function (SMD -0.18 [95% CI -0.34 to -0.01]) and five confirmed increased pain self-efficacy (SMD 0.22 [95% CI 0.02 to 0.42]). In subgroup analysis, compared with CBT, BCT-based digital interventions demonstrated their effects on pain reduction (SMD-0.23 [95% CI -0.44 to -0.03]; P=.03; I²=69%) and physical function (SMD-0.26 [95% CI -0.47 to -0.04]; P=.02; I²=60%) in the short term. In addition, physiotherapist involvement in treatment also had a positive effect on pain control (SMD-0.14 [95% CI -0.27 to -0.02]; P=.02; I²=0%) and physical function (SMD -0.16 [95% CI -0.31 to -0.01]; P=.04; I²=60%) in the short term. Furthermore, website-based digital tools improved physical function in the short term (SMD -0.21 [95% CI -0.41 to -0.01]; P=.04; I²=60%). No significant impact was observed on health-related quality of life (HRQoL) (SMD 0.12 [95% CI -0.29 to 0.53]) or physical activity (SMD 0.05 [95% CI -0.07 to 0.17]). The Grading of Recommendations, Assessment, Development, and Evaluation suggested a wide range of evidence quality, from very low to moderate.  **Conclusions**  Moderate- and low-quality evidence supported that psychotherapy-based digital tools improved pain intensity, physical function, and self-efficacy in the short term. Low- and very-low-quality evidence demonstrated uncertainty for physical activity and HRQoL or long-term effects. However, affective interactions between patients and professionals may affect clinical outcomes. |  |
| **INTRODUCTION** | | |  |
| Rationale | 3 | the increased prevalence of OA has resulted in increasing demand for therapists. To this end, remote OA rehabilitation using digital technologies is rapidly evolving to alleviate socioeconomic burden. It has been demonstrated that digitalized rehabilitation is comparable to physical therapist supervision in terms of exercise quality, physical training supervision, and sport-specific self-efficacy. Accumulating evidence has demonstrated long-term improvement in pain and physical function using digital-based rehabilitation in patients with OA . Moreover, some studies have reported that Internet-based CBT and BCT have led to significant improvement in physical activity and exercise behaviour compared with traditional treatments.  Nevertheless, it remains controversial whether digital tools can replace healthcare professionals. The efficacy of digital interventions blended with behavioural science must be thoroughly assessed before clinical implementation/application. In addition, an updated assessment is required due to rapid advances in novel digital treatments in the past decade. |  |
| Objectives | 4 | This review aimed to evaluate the efficacy of behavioural, science-based, digital interventions in patients with OA. |  |
| **METHODS** | | |  |
| Eligibility criteria | 5 | Studies fulfilling the following criteria were included in the present review and meta-analysis: randomized controlled trials (RCTs); participants included those with knee, hip, or ankle OA; comparing CBT- or BCT-based digital interventions with other treatments; and those addressing at least one component of pain, physical function, HRQoL, pain self-efficacy, or physical activity. Studies published in any language other than English and those without sufficient data were excluded. Only peer-reviewed studies involving participants ≥ 18 of age were eligible for inclusion. In this review, the term “digital intervention” refers to any solution or technology that delivers health information from healthcare providers to patients over a distance. |  |
| Information sources | 6 | The literature search was performed using the Web of Science, Embase, Cochrane Library, Ovid, and PubMed databases, from date of inception to June 27, 2023 . In addition, the reference lists of relevant reviews and selected articles were manually examined for potentially relevant/eligible trials. |  |
| Search strategy | 7 | (Supplementary Appendix 1) |  |
| Selection process | 8 | The initial search retrieved 1628 articles after removing duplicates, of which 1557 that did not fulfill the inclusion criteria were excluded. Two additional records were found, one through cross-referencing of bibliographies and the other by contacting the corresponding author(s). Subsequently, 73 eligible full-text articles were reviewed, of which 11 RCTs were ultimately included in the quantitative analysis (Figure 1). |  |
| Data collection process | 9 | All duplicate references were removed. Subsequently, titles and abstracts were manually screened for potentially eligible studies by two reviewers (DZ and HY), and relevant RCTs were identified. The full text was then retrieved by both reviewers to assess eligibility for inclusion. Disagreements between the researchers were resolved through discussion or consultation with a third reviewer (BZ). The data were cross validated by a third researcher (BZ) using EndNote 20 (Clarivate Analytics, Philadelphia, PA, USA). |  |
| Data items | 10a | The dataset comprised study information, participant characteristics, type of psychotherapy, type of digital tool, study duration, and outcome measures, including pain and function scores. If a study used multiple pain scales, the scale with the highest sensitivity to change(s) was used . |  |
|  | 10b | The function subscales of the Western Ontario and McMaster Universities Arthritis Index (WOMAC) and the KOOS/HOOS Physical Function Shortform were used to assess functional improvement. Harmonised physical function was used, with higher scores indicating more severe physical dysfunction. |  |
| Study risk of bias assessment | 11 | Risk of bias was assessed using the Risk-of-Bias Tool in the Cochrane Handbook of Systematic Evaluation. Seven domains, including random sequence generation, allocation concealment, blinding of participants and personnel, incomplete outcome data, selective reporting, and other biases, were used to evaluate quality of evidence. Each domain was assigned a judgement of low, high, or unclear risk of bias. |  |
| Effect measures | 12 | A negative SMD value for pain and physical function and a positive SMD value for HRQoL, pain self-efficacy, and physical activity favour psychotherapy-based digital tools. The magnitude of SMD was interpreted in accordance with guidelines reported by Cohen [43], as follows: SMD < 0.2 (small); 0.2 to 0.8 (medium); and > 0.8 (large). For clinical interpretation, the mean differences were calculated. |  |
| Synthesis methods | 13a | Eleven trials reported short-term effects (up to 6 months after randomisation) and six reported long-term effects (> 6 months after randomisation). Pain intensity, physical function, HRQoL, pain self-efficacy, and physical activity were evaluated in 11, eight, seven, five, and five trials, respectively (Online Supplementary Appendix 2). |  |
|  | 13b | The authors of studies with missing data were contacted. When the authors were unavailable, data were estimated using recommendations from the Cochrane Handbook (e.g., estimation of SD from standard error [SEs]). In trials in which SD was not reported, missing data were imputed from 95% confidence intervals (CIs), SEs, p-values, baseline changes, graphical representations, medians, and interquartile ranges (IQRs), or SDs from baseline. Trials in which imputations were not possible were excluded from the quantitative analysis. |  |
|  | 13c | Standardised mean differences (SMDs) were calculated to standardise the results to a uniform scale when studies assessed the same outcomes using different instruments. |  |
|  | 13d | We used meta-analysis to assess outcome effect values. If I2>50%, a random effects model was used. All analyses were performed using Review Manager version 5.4. |  |
|  | 13e | Subgroups were defined in terms of the type of psychotherapy (i.e., CBT or BCT), type of digital tool (apps, wearable devices, and phones), and the therapist involvement. |  |
|  | 13f | Sensitivity and subgroup analyses were performed to assess the potential impact of the sources of heterogeneity. To investigate the potential impact of methodological quality on the estimates, a sensitivity analysis was performed by removing one study at a time, and trials with poor methodological quality were removed. |  |
| Reporting bias assessment | 14 | Quality level in this meta-analysis was evaluated according to the Grading of Recommendations Assessment, Development, and Evaluation (GRADE) approach. Quality of evidence was classified as high, moderate, low, or very low. |  |
| Certainty assessment | 15 | Heterogeneity of the pooled studies was examined using the chi-squared test and the I^2^ statistic, with I^2^ > 50% indicating substantial heterogeneity. Publication bias was assessed by visual inspection of funnel plots and Egger’s test for meta-analyses of ≥ 10 trials. All analyses were performed using Review Manager version 5.4. |  |
| **RESULTS** | | |  |
| Study selection | 16a | The initial search retrieved 1628 articles after removing duplicates, of which 1557 that did not fulfill the inclusion criteria were excluded. Subsequently, 73 eligible full-text articles were reviewed, of which 11 RCTs were ultimately included in the quantitative analysis (Figure 1). |  |
|  | 16b | Two additional records were found, one through cross-referencing of bibliographies and the other by contacting the corresponding author(s). |  |
| Study characteristics | 17 | Ten RCTs and one cluster RCT, comprising 2108 patients, were included in the qualitative analysis. The trials were conducted in Europe (n=3 [27.3%]), Oceania (n=4 [36.4%]), and North America (n=4 [36.4%]). The sample sizes of the included trials ranged from 51 to 427; the mean (± SD) age of the patients was 62.5 ± 8.1 years, and females were predominant in the pooled population (n=1437 [65.98%]), which is consistent with the global prevalence of OA. |  |
| Risk of bias in studies | 18 | In general, the most frequent risks of bias for RCTs were incomplete outcomes (54.5%) and blinding of participants/personnel (45.5%). Other biases and selective reporting accounted for the second most frequent risks of bias (18.2%). The overall confidence in the cumulative evidence varied from very low to moderate, with low confidence being the most commonly identified (Online Supplementary Appendix 3). |  |
| Results of individual studies | 19 | (Figure 2) |  |
| Results of syntheses | 20a | (Online Supplement Appendix 6) |  |
|  | 20b | For pain reduction, 11 trials with 2108 patients reported a moderate effect of psychotherapy-based digital tools in reducing pain in the short term (SMD-0.18 [95% CI -0.33 to -0.04]; P=0.01; I²=61%), with low-quality evidence. However, in the long-term follow-up, this effect was ambiguous when six trials with 1271 patients were included (SMD -0.01 [95% CI -0.12 to 0.10]; P=0.88; I²=0%), with moderate-quality evidence. For physical function, low certainty evidence supported significant improvement in the short term (SMD -0.18 [95% CI -0.34 to -0.01]; P=.03; I²=54%) and its effect remained significant in the long-term (SMD -2.55 [95% CI -5.09 to -0.02]; P=0.05; I²=0%), with moderate-quality evidence. For HRQoL, very low-quality evidence from seven trials with 1350 patients demonstrated an uncertain effect (SMD 0.12 [95% CI -0.29 to 0.53]; P=.57; I²=92%). For pain self-efficacy, low-quality evidence from five trials with 812 patients reported a positive effect (SMD 0.22 [95% CI 0.02 to 0.42]; P=.03; I²=50%). For physical activity, moderate-quality evidence demonstrated an uncertain effect (SMD 0.05 [95% CI -0.07 to 0.17]; P=.45; I²=0%) . |  |
|  | 20c | We were unable to fully explore the reasons for heterogeneity because many covariates for psychotherapy-based digital tools in OA were not usually reported in the trials. |  |
|  | 20d | Sensitivity analysis were performed by one-study remove and sensitivity analysis revealed that the results of physical function were unstable; therefore, these results should be interpreted with caution (Online Supplement Appendix 6). |  |
| Reporting biases | 21 | Summarized according to studies, the most frequent risk of bias for randomized controlled trials was Incomplete outcome bias (54.5% studies high risk of bias) and ‘Blinding of participants/personnel’ (45.5% studies high risk of bias). Similarly, ‘other bias’ and ‘selective reporting were also the most frequent risk of bias (18.2% studies high risk of bias). Overall confidence in cumulative evidence varied from very low to moderate with low confidence most commonly identified. |  |
| Certainty of evidence | 22 | Online supplementary appendix 4 presented the GRADE evaluation of evidence |  |
| **DISCUSSION** | | |  |
| Discussion | 23a | This systematic review provides moderate to very low-quality evidence of three digital tools based on two types of psychotherapies in treating patients with OA. Behavioral change-based digital tool was proved to reduce pain intensity only at short term, and its effect on physical function could last at long-term. Among all the digital tools, Apps or website intervention and physical therapist’s involvement were proved to be effective on pain reduction and physical function with low-quality evidence. However, further high- quality trials should provide more precise estimates, mainly in the long term. |  |
|  | 23b | We were unable to fully explore the reasons for heterogeneity because many covariates for psychotherapy-based digital tool in OA are normally not recorded in trials. |  |
|  | 23c | Subgroup analyses to explore the potential impact of high risk of bias, characteristics of the population were limited by the small number of included trials or because data were poorly reported. Third, despite using extensive search techniques, evaluation may have been hampered by language bias, as we only included literature written in English in five databases. Therefore, we cannot guarantee that all eligible studies were found. Forth, this meta-analysis comprised only 11 samples, with 10 RCTs and 1 cluster RCT. Due to this rather low number of studies, Q-static is reduced in power and I2 can be biased. Finally, like other types of meta-analysis, it is prone to publication bias and other risk of bias and is limited to the information reported in the paper. |  |
|  | 23d | This study confirmed that digital tool blended with behavioral change therapy has moderate and clinical meaningful treatment effects in patients with hip, knee and hip OA at short term and long term, although the quality of evidence was very low. The findings echo the current guidelines recommending rehabilitation emphasizing exercise, self-efficacy, and self-management for patients with OA. At the age of digital technologies is rapidly evolving, the affective interaction between patient and professional is still a crucial factor, and it should be taken into account in the future studies. |  |
| **OTHER INFORMATION** | | |  |
| Registration and protocol | 24a | The present review was performed in accordance with the Preferred Reporting Items for Systematic Reviews and Meta-Analysis (i.e., “PRISMA”) and guidelines published in the Cochrane Handbook of Systematic Evaluation. The study protocol was registered with the International Register of Prospective Systematic Reviews (PROSPERO): CRD42023430716. |  |
|  | 24b | The study protocol was registered with the International Register of Prospective Systematic Reviews (PROSPERO) |  |
|  | 24c | The title switch from“Research on digital intervention for osteoarthritis based on behavioral therapy: a systematic review and meta-analysis”to “Psychotherapy-Based Digital Intervention for Treating Osteoarthritis: A Systematic Review and Meta-analysis” |  |
| Support | 25 | Non-financial support |  |
| Competing interests | 26 | No any competing interests of review authors. |  |
| Availability of data, code and other materials | 27 | Specific data can be requested from the authors, if needed: template data collection forms; data extracted from included studies; data used for all analyses; analytic code; any other materials used in the review. |  |

*From:*  Page MJ, McKenzie JE, Bossuyt PM, Boutron I, Hoffmann TC, Mulrow CD, et al. The PRISMA 2020 statement: an updated guideline for reporting systematic reviews. BMJ 2021;372:n71. doi: 10.1136/bmj.n71

For more information, visit: <http://www.prisma-statement.org/>
