# Supplementary material for: Behavioral Therapy–Based Digital Interventions for Treating Osteoarthritis: Systematic Review and Meta-Analysis
Source: J Med Internet Res. 2025 Mar 19;27:e56227. doi: 10.2196/56227 (PMC11966084; doi:10.2196/56227)

**Table S1: Search Strategy Conducted up to June 27^th^, 2023**

**PubMed**

| # | Searches | Results |
| --- | --- | --- |
| S1 | "Osteoarthritis"[Mesh]: (Osteoarthritides) OR (Osteoarthrosis) OR (Osteoarthroses) OR (Degenerative Arthritides) OR (Degenerative Arthritis) OR (Arthrosis) OR (Arthroses) OR (Osteoarthrosis Deformans) | 512,193 |
| S2 | "Behavior Therapy"[Mesh]: ((Behavior Therapies) OR (Behavior Treatment) OR (Conditioning Therapy) OR (Conditioning Therapies) OR (Behavior Change Techniques) OR (Behavior Change Technique) OR (Behavior Modification) OR (Behavior Modifications)) | 404,686 |
| S3 | "Dialectical Behavior Therapy"[Mesh]: (Dialectical Behavior Therapies) | 1080 |
| S4 | "Cognitive Behavioral Therapy"[Mesh]: ((Cognitive Behavioral Therapies) OR (Cognitive Therapy) OR (Cognitive Behaviour Therapy) OR (Cognitive Psychotherapy) OR (Cognition Therapy) OR (Cognitive Behavior Therapies) OR (Cognitive Behavior Therapy)) | 161,342 |
| S5 | S2 OR S3 OR S4 | 507,875 |
| S6 | "Telemedicine"[Mesh]: (Telemedicine) OR (Mobile Health) OR (Telehealth) OR (ehealth) OR (mhealth) | 114,253 |
| S7 | "Electronic Mail"[Mesh]: (Email) OR (E-mail) | 202,779 |
| S8 | "Mobile Applications"[Mesh]: (Smartphone) OR (smart-phone) OR (smart telephone) OR (Tablet) OR (cell) OR (hand-held) OR (Cell Phone) OR (handheld) OR (Remote Consultation) OR (Teleradiology) OR (Telenursing) OR (Computer Systems) OR (Computer-Assisted Instruction) OR (computer) OR (Digital Technologies) OR (APP) OR (Social Media) OR (Internet-Based Intervention) OR (Mobile Application) OR (Mobile App) OR (Smartphone App) OR (Portable Software Application) | 9,784,853 |
| S9 | "Internet Use"[Mesh]: (Internet) OR (web) | 314,227 |
| S10 | S6 OR S7 OR S8 OR S9 | 10,189,535 |
| S11 | S1 AND S5 AND S10 | 1,185 |
| S12 | Limit 11 to English language | 1119 |

**Web of Science**

| # | Searches |  | Results |
| --- | --- | --- | --- |
| S1 | TS= ((Osteoarthritides) OR (Osteoarthrosis) OR (Osteoarthroses) OR (Degenerative Arthritides) OR (Degenerative Arthritis) OR (Arthrosis) OR (Arthroses) OR (Osteoarthrosis Deformans)) | Search modes - Boolean/Phrase | 27,013 |
| S2 | TS= (Behavior Therapies) OR (Behavior Treatment) OR (Conditioning Therapy) OR (Conditioning Therapies) OR (Behavior Change Techniques) OR (Behavior Change Technique) OR (Behavior Modification) OR (Behavior Modifications) OR (Dialectical Behavior Therapies) OR (Cognitive Behavioral Therapies) OR (Cognitive Therapy) OR (Cognitive Behaviour Therapy) OR (Cognitive Psychotherapy) OR (Cognition Therapy) OR (Cognitive Behavior Therapies) OR (Cognitive Behavior Therapy) | Search modes - Boolean/Phrase | 3,480,346 |
| S3 | TS= (Telemedicine) OR ( Mobile Health) OR (Telehealth) OR (ehealth) OR (mhealth) OR (Email) OR (E-mail) OR (Mobile) OR (Smartphone) OR (smart-phone) OR ( smart telephone) OR (Tablet) OR (cell) OR (hand-held) OR (Cell Phone) OR (handheld) OR (Remote Consultation) OR (Teleradiology) OR (Telenursing) OR (Computer Systems) OR (Computer-Assisted Instruction) OR (Internet) OR (web) OR (computer) OR (Digital Technologies) OR (APP) OR (Social Media) OR (Internet-Based Intervention) OR (Mobile Application) OR (Mobile App) OR (Smartphone App) OR (Portable Software Application) | Search modes - Boolean/Phrase | 31,487,248 |
| S4 | S1 AND S2 AND S3 | Search modes - Boolean/Phrase | 450 |
| S5 | S1 AND S2 AND S3 | Limiters - English Language  Search modes - Boolean/Phrase | 414 |

EMBASE

| # | Searches | Results |
| --- | --- | --- |
| 1 | exp osteoarthritis/ OR exp Osteoarthritides/ OR exp Osteoarthroses/ | 191,270 |
| 2 | ‘Arthritis’:ab,ti OR ‘Osteoarthrosis’:ab,ti | 312,158 |
| 3 | 1 OR 2 | 470,979 |
| 4 | exp Behavior Therapy/ | 58,428 |
| 5 | ‘Behavior Treatment’:ab,ti OR ‘Conditioning Therapy’:ab,ti OR ‘Behavior Change Technique’:ab,ti OR ‘Behavior Modification’:ab,ti OR ‘Osteoarthrosis’:ab,ti | 9,200 |
| 6 | exp Dialectical Behavior Therapy/ | 1,789 |
| 7 | exp Cognitive Behavioral Therapy/ | 35,022 |
| 8 | ‘Cognitive Therapy’:ab,ti OR ‘Cognitive Psychotherapy’:ab,ti OR ‘Cognitive Behavior Therapy’:ab,ti | 8,652 |
| 9 | 4 OR 5 OR 6 OR 7 OR 8 | 99,782 |
| 10 | Exp Telemedicine/ | 60,386 |
| 11 | Exp Telehealth/ | 28,199 |
| 12 | Exp E-mail/ | 90,327 |
| 13 | Exp Smartphone/ | 34,683 |
| 14 | Exp teleconsultation/ | 16,130 |
| 15 | Exp ‘mobile application’/ | 23,051 |
| 16 | Exp ‘digital health’/ | 13,822 |
| 17 | Exp teleradiology/ | 2,357 |
| 18 | Exp telenursing/ | 566 |
| 19 | Exp computer system/ | 30,587 |
| 20 | ‘Internet’:ab,ti OR ‘web’:ab,ti OR ‘computer’:ab,ti OR ‘Digital Technologies’:ab,ti OR ‘Social Media’:ab,ti OR ‘Portable Software Application’:ab,ti OR ‘Mobile Health’:ab,ti OR ‘ehealth’:ab,ti OR ‘mhealth’:ab,ti OR ‘Computer-Assisted Instruction’:ab,ti | 647,221 |
| 21 | 10 OR 11 OR 12 OR 13 OR 14 OR 15 OR 16 OR 17 OR 18 OR 19 | 853,144 |
| 22 | 3 AND 9 AND 21 | 83 |
| 23 | limit 22 to English language | 78 |

Cochranelibrary

| # | search | results |
| --- | --- | --- |
| 1 | (Osteoarthritides) OR (Osteoarthrosis) OR (Osteoarthroses) OR (Degenerative Arthritides) OR (Degenerative Arthritis) OR (Arthrosis) OR (Arthroses) OR (Osteoarthrosis Deformans) :ti,ab,kw | 1,561 |
| 2 | ((Behavior Therapy) OR (Behavior Treatment) OR (Conditioning Therapy) OR (Conditioning Therapy) OR (Behavior Change Technique) OR (Behavior Modification) OR (Dialectical Behavior Therapy) OR (Cognitive Behavioral Therapy) OR (Cognitive Therapy) OR (Cognitive Behaviour Therapy) OR (Cognitive Psychotherapy) OR (Cognition Therapy) OR (Cognitive Behavior Therapy)) | 89,903 |
| 3 | ((Telemedicine) OR ( Mobile Health) OR (Telehealth) OR (ehealth) OR (mhealth) OR (Email) OR (E-mail) OR (Mobile) OR (Smartphone) OR (smart-phone) OR (smart telephone) OR (Tablet) OR (cell) OR (hand-held) OR (Cell Phone) OR (handheld) OR (Remote Consultation) OR (Teleradiology) OR (Telenursing) OR (Computer Systems) OR (Computer-Assisted Instruction) OR (Internet) OR (web) OR (computer) OR (Digital Technologies) OR (APP) OR (Social Media) OR (Internet-Based Intervention) OR (Mobile Application) OR (Mobile App) OR (Smartphone App) OR (Portable Software Application)):ft | 106,654 |
| 3 | ((Telemedicine) OR ( Mobile Health) OR (Telehealth) OR (ehealth) OR (mhealth) OR (E-mail) OR (Mobile) OR (Smartphone) OR (Tablet) OR (hand-held) OR (Cell Phone) OR (handheld) OR (Remote Consultation) OR (Teleradiology) OR (Telenursing) OR (Computer Systems) OR (Computer-Assisted Instruction) OR (Internet) OR (web) OR (computer) OR (Digital Technologies) OR (APP) OR (Social Media) OR (Internet-Based Intervention) OR (Mobile Application) OR (Mobile App) OR (Smartphone App) OR (Portable Software Application)) :ft | 205,782 |
| 4 | #1 AND #2 AND #3 | 63 |
| 5 | limit 4 to English language | 63 |

Ovid

| # | search | results |
| --- | --- | --- |
| 1 | osteoarthritis.mp. or exp Osteoarthritis/ [mp=title, abstract, heading word, drug trade name, original title, device manufacturer, drug manufacturer, device trade name, keyword, floating subheading word, candidate term word] | 112,549 |
| 2 | Arthritis/ or Arthritis.mp. [mp=title, abstract, heading word, drug trade name, original title, device manufacturer, drug manufacturer, device trade name, keyword, floating subheading word, candidate term word] | 253,219 |
| 3 | 1 OR 2 | 343,041 |
| 4 | Behavior Therapy.mp. or Behavior Therapy/ [mp=title, abstract, heading word, drug trade name, original title, device manufacturer, drug manufacturer, device trade name, keyword, floating subheading word, candidate term word] | 34,844 |
| 5 | cognitive behavioral therapy/ or cognitive remediation/ or dialectical behavior therapy/ | 31,230 |
| 6 | 4 OR 5 | 62,603 |
| 7 | mobile health.mp OR tele referral.mp OR telehealth.mp OR telemedicine.mp OR ehealth.mp OR mhealth.mp. [mp=title, abstract, heading word, drug trade name, original title, device manufacturer, drug manufacturer, device trade name, keyword, floating subheading word, candidate term word] | 69,612 |
| 8 | electronic mail/ or cell phone/ | 12,861 |
| 9 | Remote Consultation/ or Telenursing/ or Teleradiology/ | 7,134 |
| 10 | computer systems/ or software/ | 136,957 |
| 11 | Internet-Based Intervention/ or Internet/ | 82,690 |
| 12 | mobile applications/ or web browser/ | 13,045 |
| 13 | 7 OR 8 OR 9 OR 10 OR 11 OR 12 | 335,552 |
| 14 | 3 AND 6 AND 13 | 29 |
| 15 | limit 14 to English language | 29 |

**Table S2** Demographic data of study participants (n=10)

| Author | Year | research purpose | Average age | Sex Female（n%） | Affected joint | | |
| --- | --- | --- | --- | --- | --- | --- | --- |
|  |  |  |  |  | knee | hip | all |
| Bennell [36] | 2020 | To assess whether a 24-week SMS intervention improves adherence to unsupervised home exercise in patients with knee OA and obesity compared with no SMS | 61.7 (6.7) | 74（67.2%） | ✓ |  |  |
| Rini [48] | 2015 | Potential efficacy and acceptability of an Internet-based version of PCST | 67.62 (9.45) | 91 (81%) |  |  | ✓ |
| Hinman [49] | 2020 | Evaluating exercise advice and support interventions for people with osteoarthritis of the knee in a physiotherapist-led telephone call. | 62.5 (8.1) | 110(63%) | ✓ |  |  |
| McCurry [50] | 2021 | To evaluate the effectiveness of telephone CBT-I versus educational only control (EOC) in older adults with moderate to severe osteoarthritis pain. | 70.2 (6.8) | 244(74.6%) |  |  | ✓ |
| Pelle [26] | 2020 | Evaluating the short-term impact of using the dr.Bart app on the number of secondary medical consultations and clinical outcomes in Dutch patients with knee/hip osteoarthritis | 62.1 (7.7) | 306 (71.7%) |  |  | ✓ |
| Mecklenburg [51] | 2018 | Evaluating the efficacy of a remotely delivered digital care program for chronic knee pain. | 46 (12) | 57 (37%) | ✓ |  |  |
| Nelligan [24] | 2021 | Effects of an automated text message-supported web-based self-enhancement exercise and physical activity program for patients with osteoarthritis of the knee | 60 (8.4) | 109(61%) | ✓ |  |  |
| Bennell [35] | 2017 | Investigating Whether Synchronous Telephone Coaching Improves the Clinical Effectiveness of Physical Activity Programs Developed by Physical Therapists for OA of the Knee at Home | 61.1 (6.9) | 106(63.09%） | ✓ |  |  |
| Kloek [52] | 2018 | Investigating the effectiveness of a mixed physiotherapist intervention for patients with arthritis | 63.8 (8.5) | 141（67.8%） |  |  | ✓ |
| Li [53] | 2020 | Examining the Impact of a 12-Week Multifaceted Wearable Program on Physical Activity and Patient Prognosis in Patients with KOA | 65.0 (8.3) | 65（84.4%） | ✓ |  |  |

**Table S3** Mechanisms for implementing the research intervention

| Author | behavioral therapy | Method | | | | | Professional support | Timing | Inter-vention period | Adherence |
| --- | --- | --- | --- | --- | --- | --- | --- | --- | --- | --- |
|  |  | Telephone  /SMS | RMON | app | wearable device | Website | face-to-face treatment |  |  |  |
| Bennell [36] | BCT | ✓ |  | ✓ |  |  | ✓ | three times a week | 12 weeks | ✓ |
| Rini [48] | CBT | ✓ |  | ✓ |  |  |  | One module per week | 9-11 weeks |  |
| Hinman [49] | BCT | ✓ |  |  |  | ✓ |  | 5-10 consultations per month | 24 weeks |  |
| McCurry [50] | CBT | ✓ |  |  |  |  |  | 6 times in 8 weeks | 8 weeks |  |
| Pelle [26] | BCT |  |  | ✓ |  |  |  | Get daily push notifications | 24 weeks |  |
| Mecklenburg [51] | CBT | ✓ |  | ✓ | ✓ |  | ✓ | three times a week | 12 weeks |  |
| Nelligan [24] | BCT | ✓ |  |  |  | ✓ |  | 2.5 messages per week | 24 weeks | ✓ |
| Bennell [35] | CBT | ✓ |  |  |  | ✓ |  | 6 times during treatment | 18 months | ✓ |
| Kloek [52] | BCT | ✓ |  | ✓ | ✓ | ✓ | ✓ | 2 times in 3 days | 12 weeks | ✓ |
| Li [53] | BCT | ✓ | ✓ |  | ✓ |  | ✓ | Twice a week | 12 weeks |  |

**Table S4** Functions of digital tools

|  | education | target setting | action plan | Incentives | Reminder | Self  supervise | Publishing experience | exercise component | | | |
| --- | --- | --- | --- | --- | --- | --- | --- | --- | --- | --- | --- |
|  |  |  |  |  |  |  |  | aerobics | sleep | Balance | Physical activity |
| Bennell [36] |  |  | ✓ |  | ✓ |  |  |  |  |  | ✓ |
| Rini [48] | ✓ | ✓ | ✓ |  | ✓ | ✓ | ✓ | ✓ |  |  | ✓ |
| Hinman [49] |  | ✓ | ✓ |  | ✓ |  |  |  |  |  | ✓ |
| McCurry [50] | ✓ |  |  |  | ✓ |  |  |  | ✓ |  |  |
| Pelle [26] | ✓ | ✓ | ✓ | ✓ |  | ✓ |  |  |  |  | ✓ |
| Mecklenburg [51] | ✓ |  | ✓ |  |  | ✓ |  | ✓ |  |  | ✓ |
| Nelligan [24] | ✓ | ✓ | ✓ |  |  |  |  |  |  |  | ✓ |
| Bennell [35] | ✓ |  |  |  | ✓ | ✓ |  | ✓ |  |  | ✓ |
| Kloek [52] | ✓ | ✓ | ✓ |  | ✓ | ✓ |  |  |  |  | ✓ |
| Li [53] |  |  | ✓ |  | ✓ | ✓ |  |  |  |  | ✓ |

**Figure S1.** Funnel plot for pain


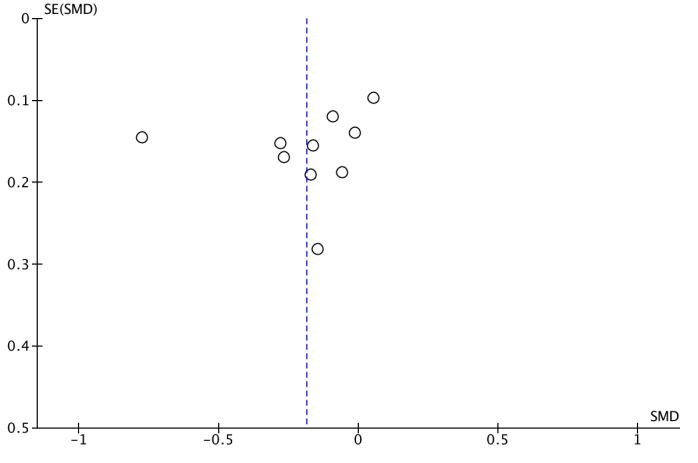

Supplement: Multimedia Appendix 2 [file jmir_v27i1e56227_app2.docx]
